# Trace Metal(loid) Migration from Road Dust to Local Vegetables and Tree Tissues and the Bioaccessibility-Based Health Risk: Impacts of Vehicle Operation-Associated Emissions

**DOI:** 10.3390/ijerph20032520

**Published:** 2023-01-31

**Authors:** Guangbo Liu, Tian Chen, Jinli Cui, Yanping Zhao, Zhi Li, Weixin Liang, Jianteng Sun, Zhenghui Liu, Tangfu Xiao

**Affiliations:** 1 Key Laboratory for Water Quality and Conservation of the Pearl River Delta, Ministry of Education, School of Environmental Science and Engineering, Guangzhou University, Guangzhou 510006, China; 2 Guangdong Provincial Key Laboratory of Petrochemical Pollution Processes and Control, School of Environmental Science and Engineering, Guangdong University of Petrochemical Technology, Maoming 525000, China; 3 Department of Civil and Environmental Engineering, The Hong Kong Polytechnic University, Hung Hom, Kowloon, Hong Kong; 4 Guangdong Provincial Key Laboratory of Chemical Measurement and Emergency Test Technology, Institute of Analysis, Guangdong Academy of Sciences (China National Analytical Center, Guangzhou), Guangzhou 510070, China

**Keywords:** bioaccessibility, chemical sequential extraction, health risk assessment, road dust, trace metal(loid) contamination, vehicle emissions

## Abstract

Traffic activities release large amounts of trace metal(loid)s in urban environments. However, the impact of vehicle operation-associated emissions on trace metal(loid) enrichment in road dust and the potential migration of these trace metal(loid)s to the surrounding environment remain unclear. We evaluated the contamination, sequential fraction, and bioaccessibility of trace metal(loid)s in urban environments by assessing their presence in road dust, garden vegetables, and tree tissues, including bark and aerial roots, at a traffic-training venue impacted by vehicle operation emissions and, finally, calculated the bioaccessibility-based health risk. The results indicated a significant accumulation of trace metal(loid)s in road dust, with the highest lead (Pb), cadmium (Cd), and antimony (Sb) concentrations in the garage entrance area due to higher vehicle volumes, frequent vehicle starts and stops, and lower speeds. Aerial roots exposed to hill start conditions exhibited the highest Pb, Zn, and Sb levels, potentially caused by high road dust resuspension, confirming that this tree tissue is an appropriate bioindicator. Sequential extraction revealed high percentages of carbonate-, Fe/Mn oxide-, and organic/sulphide-associated fractions of Pb, copper (Cu), and zinc (Zn) in road dust, while most Cd, Cr, Ni, and Sb occurred as residual fractions. According to the potential mobilizable fractions in sequential extraction, the in vitro gastrointestinal method could be more suitable than the physiologically based extraction test to evaluate the bioaccessibility-related risk of traffic-impacted road dust. The bioaccessibility-based health risk assessment of the road dust or soil confirmed no concern about noncarcinogenic risk, while the major risk originated from Pb although leaded gasoline was prohibited before the venue establishment. Furthermore, the cancer risks (CRs) analysis showed the probable occurrence of carcinogenic health effects from Cd and Ni to adults and from Cd, Cr, and Ni to children. Furthermore, the Cd and Pb concentrations in the edible leaves of cabbage and radish growing in gardens were higher than the recommended maximum value. This study focused on the health risks of road dust directly impacted by vehicle emissions and provides accurate predictions of trace metal(loid) contamination sources in the urban environment.

## 1. Introduction

Recent rapid urbanization in developing countries has resulted in the release of large amounts of trace metal(loid)s in urban environments from industrial and traffic activities [1,2]. Consequently, urban soil and surface dust have been heavily contaminated by trace metal(loid)s, posing severe health risks to residents, particularly children [3].

Traffic-related activities have become the dominant source of trace metal(loid) contamination in road dust and topsoil in global megacities [2,4,5], particularly in the case of Pb, Cu, Zn, and Cd [6,7,8]. Since the prohibition of leaded gasoline, nonexhaust vehicle-emission sources have attracted increasing attention and contribute to nearly half of the trace metal(loid)s in urban areas [9,10,11]. The sources of well-known traffic-related trace metal(loid) contamination in road dust have generally been identified: Cu and Zn originate from deceleration operations (brake lining) and tire wear, Pb and Cd result from petrol combustion residues, and Cr and Ni stem from brake and tire wear [12,13]. A recent and more specific analysis indicated very high concentrations of Cu, Zn, Sb, etc. in tire wear powder [14]. In addition to the materials related to vehicle operation, non-negligible road materials, particularly asphalt, contain significantly high Pb and Zn concentrations [14], complicating the source identification of trace metal(loid)s and the formulation of traffic-associated contamination control strategies.

Compared to Pb, Zn, and Cu, the contributions of Cr and Ni released from braking operations and tire wear to carcinogenic risk have substantially increased in the rapidly developing megacity, Shenzhen [9]. Recently, highly toxic elements, particularly Sb, have been reported more frequently in road dust; such elements can accumulate and eventually reach concentrations that are 4–10 times the background levels [15,16]. The significantly increased amounts of Sb in road dust and nearby soil urge a further risk assessment of traffic-contaminated matrices [17,18]. However, the potential health risks associated with Sb that originates from specific vehicle emissions and is found in road dust, surface soil, and plants remain unclear.

Urban trace metal(loid) contamination poses a critical threat to living organisms, including plants and humans. Vegetables and trees, widely found in city roadside areas, are exposed to cumulative traffic pollution; furthermore, plant leaves and tree bark are suitable short-term (several months) and relatively long-term (more than one year) bioindicators, respectively [19,20,21]. Trace metal(loid)s originating from urban soil and roadside dust can passively accumulate in the residents of surrounding areas, particularly children, via direct ingestion or inhalation [22,23,24]. The probable absorbed fractions of soil and road dust cannot be accurately estimated based on the total amount of trace metal(loid)s because some immobile fractions are not bioaccessible [25]. Recently, bioaccessibility assessments of urban soil and other contaminated soils have been performed in many studies to evaluate the health risks of trace metal(loid)s [23,26,27]. Currently, a few studies have investigated the potential bioaccessibility-based health risks of trace metal(loid)s in street dust [4,5,28,29,30,31,32]. However, difficulties were found in identifying the impact of nonexhaust vehicle emissions on trace metal(loid) bioaccessibility in road dust due to the presence of road construction materials [14].

Therefore, it is necessary to investigate the trace metal(loid) enrichment, chemical fraction, and bioaccessibility in road dust impacted by specific vehicle-related emissions. In this study, we aimed to thoroughly evaluate the trace metal(loid) geochemistry and health risks of vehicle operation emissions considering road dust, garden vegetables, and local trees within a specific ecological loop at a traffic-training venue. The site has been in operation for almost 10 years, and the training road was built using local soil subjected to simple hardening without typical road materials, including asphalt. This study provides crucial information on the environmental assessment of traffic-associated contamination and could be helpful for further land reutilization.

## 2. Materials and Methods

### 2.1. Study Area and Sample Collection

**Figure 1:**
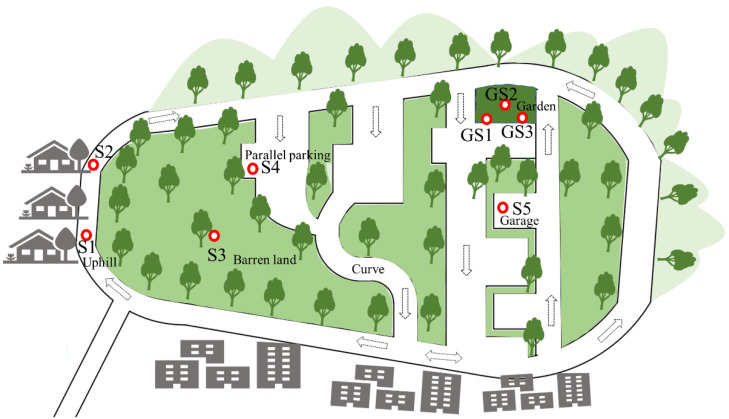
Sampling distribution at the traffic-training venue. Around the venue, two sides contain hills with abundant trees and grass, and the other two sides contain buildings. The five sampling locations were #S1, located at the entrance of the venue, representing hill start (HS) conditions showing rapid speeds and frequent start and stop operations; #S2, representing driving conditions after hill starts; #S3, barren land receiving airborne dust depositions from #S1, #S2, and curve driving; #S4, representing parallel parking; and #S5, representing driving back to the garage showing low speeds and frequent start and stop operations. Road dust (RD1–5), aerial roots, and tree bark were collected at each sampling location. Three garden soil (GS) samples were collected: radish (GS1) and tomato (GS3) were located near the roadside, while cabbage (GS2) was grown in the center of the garden.

The samples were collected in December 2018 at a training venue in Guangzhou, southern China. A subtropical marine monsoon climate prevails in the region, showing an average monthly temperature of 10.8–21 °C, wind primarily from the north within 0.2–8.7 miles per hour, precipitation of 29.4–67.9 mm, and humidity of 67–69% in winter [33]. The training venue had been in operation for approximately 10 years, long after the ban of leaded gasoline in 1997, and is currently under environmental evaluation before primary school construction. The site includes one entrance and vehicles are driven on a closed loop; traffic-related emissions are the only anthropogenic source of trace metal(loid) contamination. The two sides of the venue are surrounded by hills with abundant trees and grass; the other two sides are surrounded by buildings and the venue entrance is located in the corner (Figure 1). The sampling locations included #S1, located at the entrance of the venue, representing hill start conditions with rapid speed and frequent start and stop vehicle operations; #S2, representing driving conditions after hill starts with a smooth traffic flow; #S3, barren land receiving airborne dust depositions from #S1, #S2, and curve driving; #S4, representing parallel parking; and #S5, representing driving back to the garage with low speed and frequent start and stop vehicle operations. 

At each location (Figure 1), a series of samples were collected, including road dust (RD) and plant samples, following our previous studies [12,32]. Surface road dust particles were collected using a brush and dustpan in the absence of precipitation for more than seven days. Garden vegetables have been grown near site #S5 since the start of the driving school operation, and three garden soil samples (GS1–3) with vegetables were collected to determine the impact of dust accumulation on trace metal(loid) enrichment in soil and plants. The garden soil (GS) was located near #S5; radish (GS1) and tomato (GS3) were grown near the roadside, and cabbage (GS2) was grown in the center of the garden (Figure 1). 

Dust can be easily suspended into air due to traffic activities and wind impact, from where it can be taken up by plants and ingested by humans [34,35]. Banyan trees, prevalent in southern China and around the world, can indicate urban contamination conditions via their leaves [12] and possibly via tree bark and aerial roots. Aerial roots and outer bark were carefully sampled using stainless steel cutters, and the inner bark (epidermis layer) was sampled after removing the outer bark layers. To maintain consistency, the height of the tree samples ranged from 1.5 to 2 m. Aerial root samples were also collected in a control park. The control park, where vehicles are prohibited, is a background site located 5 km away from the study site. Abundant trees and grass inside and around the park can inhibit the entry of a large portion of outside dust into the park. The samples were placed in polyethylene bags, transferred to the laboratory, and subsequently cut with ceramic scissors to prevent contamination. 

### 2.2. Sample Preparation and Analysis

The soil and road dust samples were air-dried in the laboratory, crushed, and sieved through a 2 mm polyethylene sieve to remove stones, coarse materials, and other debris. The tree and vegetable samples were thoroughly washed with tap and deionized (DI) water and dried in an oven at 60 °C for 3 days. All the samples were ground into powder using a clean ball mill (ZQM-P2, Chansha Miqi, China) and stored in the dark before analysis.

Trace metal(loid)s in the soil, road dust, and plant samples were subjected to pseudo-total digestion using HNO_3_, HCl, HF, and HClO_4_ in polytetrafluoroethylene (PTFE) digestion tubes (50 mL) [32], after which they were analyzed via MP-AES (4100, Agilent) or ICP-MS (8800, Agilent) and reported on a dry weight basis. The detection limits of ICP-MS for soil, dust, and plant samples were 3.5 μg/L for Fe, 0.5 μg/L for Al, 0.1 μg/L for Pb, 0.002 μg/L for Cd, 0.2 μg/L for Cr, 0.04 μg/L for Ni, 0.03 μg/L for Cu, 0.07 μg/L for Zn, 0.02 μg/L for As, 0.6 μg/L for Sb, 0.07 μg/L for Ba, and 0.05 μg/L for Mn. The detection limits of the MP-AES were 10.8 μg/L for Fe and 3.2 μg/L for Al, which were used for soil and dust analysis. The operating details of the ICP-MS and MP-AES are provided in the supplementary file. CaCl_2_-extractable elements in road dust and garden soil were determined in filtered (0.45-μm) extracts by mixing dust/soil with a CaCl_2_ (0.01 M) solution (1/10, g/mL) for 120 min [36]. Plant-available trace metal(loid)s in soil were evaluated using a rhizosphere-based extraction method (1/10, g/mL) involving a combined mixture of 10 mM organic acids (acetic, lactic, citric, malic, and formic acid at a molar ratio of 4:2:1:1:1) in the dark for 16 h [36].

The five operational sequential fractions were analyzed according to a previous study [37] with slight modifications [38] and were described as follows: (1) exchangeable fraction (F1, 0.5 M MgCl_2_), (2) carbonate-bound and specifically adsorbed fraction (F2, acid-extractable part, 1 M CH_3_COONa), (3) Fe/Mn oxide fraction (F3, reducible part, 0.04 M NH_2_OH·HCl), (4) organic/sulphide fraction (F4, oxidizable part, 30% H_2_O_2_ and 3.2 M CH_3_COONa in 20% HNO_3_), and (5) residual fraction (F5), which was obtained by subtraction from the pseudo-total digestion result.

The anthropogenic contribution of trace metal(loid)s (M) in the road dust and soil samples was evaluated by calculating the enrichment factor (EF) using the equation EF = (M/Al)_sample_/(M/Al)_UCC_, where the aluminum (Al) and upper continental crust (UCC) values were considered for normalization [39]. The contamination levels were classified as follows: EF < 2, minimal enrichment; 2 < EF < 5, moderate enrichment; 5 < EF < 20, significant enrichment; 20 < EF < 40, very high enrichment; and EF > 40, extremely high enrichment [40].

Although the uptake of trace metal(loid)s by plant aerial roots and outer roots from underground soil cannot be eliminated, we calculated the ratio of trace metal(loid) concentrations in the plant samples to those in the road dust samples as the bioconcentration factor (BCF) to evaluate possible transferability or accumulation [41].

### 2.3. Bioaccessibility Assessment of Trace Metal(loid)s in Road Dust and Soil

Two typical bioaccessibility test methods, the in vitro gastrointestinal (IVG) method and physiologically based extraction test (PBET), were employed to investigate the potential mobilizable fractions of trace metal(loid)s in road dust in simulated gastric and intestinal solutions once ingested [23,42]. For the gastric-phase extraction, triplicate samples were accurately weighed in a gastric phase fluid for the IVG (1 g in 150 mL, pH 2.5) or PBET (1 g in 100 mL, pH 1.8) analysis and agitated horizontally at 150 rpm at 37 °C; the detailed composition of the gastric and intestinal solution can be found in Table S1 in the Supplementary Information. The suspensions were then centrifuged at 4000 rpm for 10 min, and 10% of the gastric-phase supernatant was collected for analysis. The remainder of the gastric-phase solutions was adjusted to pH 5.5 (IVG) or pH 7.0 (PBET) with Na_2_CO_3_, and bile and pancreatin were subsequently added for the intestinal-phase extraction. All the suspensions were centrifuged, filtered through a 0.22 μm filter, and digested prior to the analysis. The bioaccessibility (BA%) of trace metal(loid)s was calculated by dividing the bioaccessible fraction, including the gastric and intestinal phases, by the total content in dust or soil.

### 2.4. Human Health Risk Model

The exposure health risks of trace metal(loid)s in road dust to the human body was determined using the human health evaluation method developed by the US Environmental Protection Agency [43,44]. The chronic daily intake (CDI, i.e., the average daily dose or ADD, mg/kg/day) of bioaccessible trace metal(loid)s via incidental ingestion of road dust and garden soil [26,45] was calculated as follows: CDI = C_sample_ × BA × ExFre × ED × IR/(BW × AT) × 10^−6^. The details of the equation parameters can be found in Table S2 in the Supplementary Information.

The noncarcinogenic risk of trace metal(loid)s in road dust was estimated by the hazard quotient (HQ) using the CDI divided by the reference toxicity value [46] as follows: HQ = CDI/RfD, where RfD is the reference dose. The hazard index (HI) is equal to the sum of the HQ values for all the trace metal(loid)s, i.e., HI = HQi. An HI value higher than one suggests the possible occurrence of noncarcinogenic effects, i.e., any adverse toxic effects but not cancer health effects, and a higher HI value indicates a higher probability [26,43,47]. 

The lifetime carcinogenic hazard was calculated by the cancer risk (CR) using the CDI and slope factor (SF): CR = CDI × SF. An SF value was used to assess the carcinogenic risk due to lifetime exposure to trace metal(loid)s. A CR value lower than 10^−6^ (a probability of 1 in 1,000,000 of an individual developing cancer) was considered negligible (acceptable), while a CR value higher than 10^−4^ (a probability of 1 in 10,000 of an individual developing cancer) was considered unacceptable [48]. The CR value 10^−6^ is also suggested as the carcinogenic target risk by USEPA [48].

### 2.5. Quality Assurance/Quality Control and Statistical Analysis

Quality assurance/quality control during digestion were performed using standard reference materials, including GBM908-10, OREAS-45e, and GBW10048. The recovery rates of all the elements in these standard materials remained within 89.6–121.4%. Randomly selected samples corresponding to one-tenth of the total number were subjected to triplicate analyses; the results indicated a bias ranging from 0.5–7.7% for all the metal(loid)s, while the bias for Cr remained within 12.6%; the average values of trace metal(loid)s are reported. The detection limits of ICP-MS for soil, dust, and plant samples were 3.5 μg/L for Fe, 0.5 μg/L for Al, 0.1 μg/L for Pb, 0.002 μg/L for Cd, 0.2 μg/L for Cr, 0.04 μg/L for Ni, 0.03 μg/L for Cu, 0.07 μg/L for Zn, 0.02 μg/L for As, 0.6 μg/L for Sb, 0.07 μg/L for Ba, and 0.05 μg/L for Mn. The detection limits of MP-AES were 10.8 μg/L for Fe and 3.2 μg/L for Al, which were used for the soil and dust analysis. Pearson’s correlation analysis was performed with the SPSS 17.0 software.

## 3. Results and Discussion

### 3.1. Trace Metal(loid)s in Road Dust and Garden Soil

The metal concentrations in the road dust and garden soil samples retrieved from the training venue and noncontaminated background park are summarized in Table 1. Generally, the trace metal(loid)s in the road dust samples exhibited decreasing average concentrations in the following order: Zn > Pb > Cu > Cr > Ni > As > Sb > Cd. The trace metal(loid) concentrations in the dust samples substantially exceeded the background values for soils in the Guangdong Province and the levels in the dust samples retrieved from the control park. Compared to other reports of road dust in Guangzhou, the metal concentrations at the training venue generally remained within the observed ranges [12,24,49]. 

Traffic volume and speed notably impact the heterogeneous distribution of trace metal(loid) contamination in road dust [12]. At the study site (Figure 1), sampling site #S5 was strongly influenced by a higher traffic volume and lower speed as it represents a location where vehicles frequently start and stop. The analysis results (Table 1) indicated the highest concentrations of Zn, Pb, and Cu in road dust at 818.0, 282.0, and 172.5 mg/kg, respectively. Consistently, the highest concentrations of Sb (8.64 mg/kg), Cr (128 mg/kg), and Ni (25.3 mg/kg) were observed in road dust collected at site #S5. Road dust at #S2, where traffic flowed smoothly after descending the steep slope (Figure 1), showed the lowest concentrations of Sb, Zn, Cu, Cr, and Ni (Table 1). Therefore, the trace metal(loid) distributions at the site suggested that the influence of traffic on metal contamination in surface soil/dust largely depended on vehicle transport operation conditions, particularly frequent start operations, which directly impact brake pad lining, tire wear, and gasoline combustion [51]. The results eliminated the influence of road construction materials and provided an accurate evaluation of the impact of vehicle operation on trace metal(loid) accumulation in road dust.

**Figure 2:**
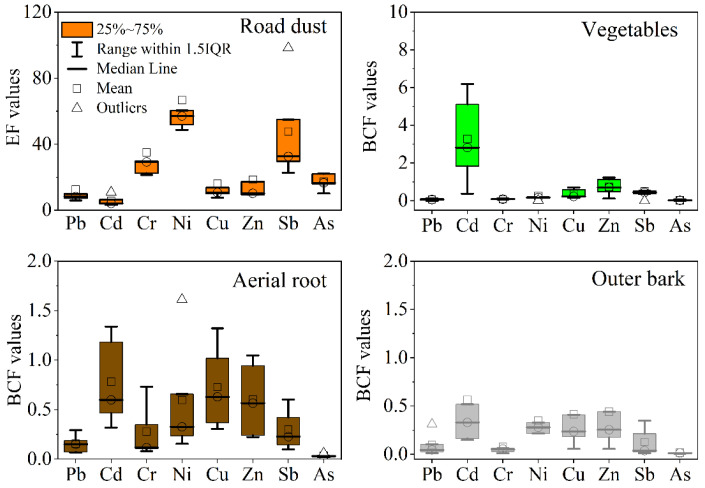
Enrichment factor (EF) values for trace metal(loid)s in road dust and bioaccumulation factor (BCF) values for trace metal(loid)s in vegetables and aerial roots and the outer bark of banyan trees.

On the basis of comparison with the background values, the calculated EF values indicated that the top five contaminants in road dust followed the general order: Ni (48.7–115.3) > Sb (22.8–98.4) > Cu (7.6–39.3) > Zn (9.4–46.6) > Pb (5.9–32.1) > Cd (3.2–10.9) (Figure 2). The EF results revealed that the site showed significant to extremely high enrichment of these trace metal(loid)s. Extremely high EF values for Sb and Ni are unexpected at traffic-training venues. The high Sb concentrations were 3.8–12.9 times those in the control samples and the background values for Guangzhou soil (Table 1), and to our knowledge, this is the first report of the Sb content in traffic-associated dust in the region. Due to its potential toxicity, Sb may pose a health risk to humans in urban regions. The emerging metal contaminant Sb mainly originates from vehicle brake systems because Sb is found in automobile brake pads [7,16,18]. Nickel, Pb, Cu, and Zn have been reported to be typical traffic activity-associated contaminants and could be the most relevant indicators of traffic contamination [7,12,16]. Although the site had been in operation long after the prohibition of Pb-containing petrol use, Pb-free petrol may still lead to contamination of Pb in dust and surface soil [52]. Furthermore, traffic activities, including tire and brake lining wear, still emit large amounts of Pb into the environment [52,53]. The high Cu and Zn contents in the surface dust samples originated from brake abrasion and exhaust fumes [1,53], consistent with the observed black particles resulting from tire abrasion and debris in the road dust samples. It was reported that high Cd concentrations at busy traffic sites may be caused by tire wear, which produces particles that usually contain significant amounts of Cd [5,16].

Similar to the road dust samples, the garden soil samples were highly contaminated by Zn, Pb, Cu and other trace metal(loid)s, with much higher levels than those in underground soil at depths from 20 to 25 cm (Table 1). The three garden soil samples exhibited Pb, Cu, and Zn levels exceeding the risk screening values in the Chinese risk control standards for soil contamination (GB 15618-2018) [54], while the Cd content (0.35 mg/kg) at GS1 slightly exceeded the standard (0.30 mg/kg), indicating that the soil merited further monitoring. Considering the sampling distribution, the trace metal(loid)s in these three soil samples exhibited the order GS1 > GS2, reflecting that soil closer to the road typically received more road dust particles (Figure 1). These results reflected the direct impact of traffic-related emissions and concerns about the quality of vegetables or crops grown near roads with a high traffic volume.

**Figure 3:**
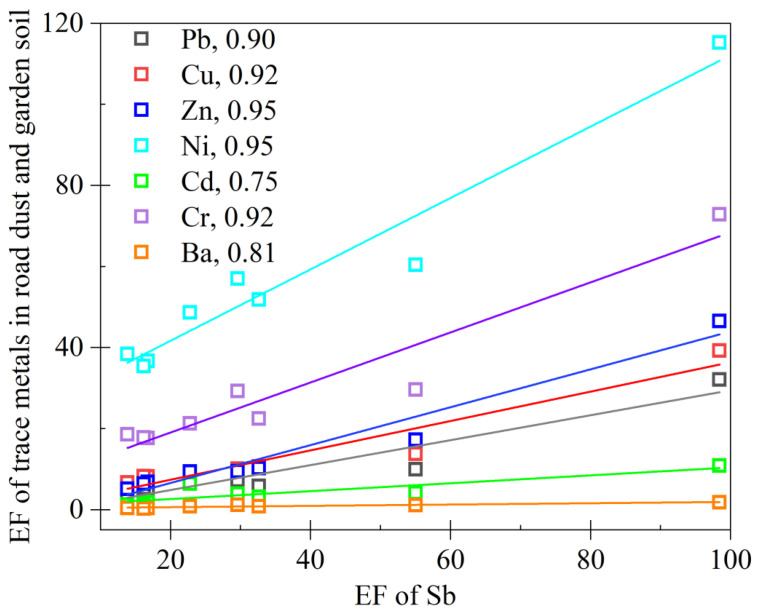
Relationship (*p* < 0.01) of enrichment factors (EFs) for Sb and Cu, Zn, Ba, Cr, Cd, Ni, and Pb in road dust and garden soil, and R^2^ data were provided after each element. The EFs are normalized by Al (aluminum) using the upper continental crust (UCC) as a reference [39].

The calculated EF values indicated a dominant anthropogenic influence on the accumulation of Sb, Cu, Zn, and Pb [55]. Noteworthy correlations of the EF values between Sb and Cu, Zn, Cd, Cr, and Ba were obtained at the training site (Figure 3), revealing similar results with very high R^2^ values between 0.75 and 0.95. The results supported the existence of one dominant source of these elements at the training site. Considering the dominant vehicle training activities and the very high EF values of the trace metal(loid)s, the dominant source should be traffic emissions, including brake pad and tire wear [56]. High levels of Cr mainly originated from the high frequency of gasoline engine exhaust emissions in the training process [53]. High concentrations of Ba were observed in brake-derived particles, consistent with brakes being the dominant source of Ba in urban environments [57,58]. The braking process was also identified as a crucial primary urban source of trace metal(loid) contamination in road dust in a Caribbean industrial city [55] and in atmospheric particulate matter (PM_10_) in London [58].

In comparison with the background value in Guangzhou [50] and the deep soil sample (20–25 cm) in the venue (Table 1), the relatively low As contamination levels in both the road dust and soil samples at the study site indicated slight contamination resulting from traffic-related emissions [32,59], likely dominated by lithogenic sources [60]. 

### 3.2. Trace Metal(loid) Accumulation in Vegetables, Aerial Roots, and Bark

Trace metal(loid) concentrations in the plant samples are shown in Table 1. The collected radish, cabbage leaf, and tomato fruit are the edible part, while tomato leaf is the non-edible residue in the field. The leaves of vegetables (cabbage, radish, and tomato) grown in the garden soil at the training venue exhibited the following values: 0.64–1.61 mg/kg Cd, 4.3–13.0 mg/kg Pb, 13.2–52.8 mg/kg Cu, 111–322 mg/kg Zn, 0.84–2.41 mg/kg As, 1.18–3.61 mg/kg Sb, and 5.1–7.7 mg/kg Cr (Table 1). Among these metal(loid)s, Cd had the highest BCF values; the other metal(loid)s followed the order Zn < Cu < Sb ≈ Pb ≈ Ni ≈ Cr ≈ As (Figure 2). Compared to the permissible safe values of contaminants in vegetables in China [61], the Cd and Pb contents in cabbage and radish exceeded the maximum levels (0.05 mg/kg for Cd and 0.3 mg/kg for Pb), and these vegetables could be considered not edible. Very few studies have reported trace metal(loid) concentrations in vegetables grown near roadsides, but reports have indicated possible contamination of Cd and Pb in lettuce from urban horticulture [62] and *Amaranthus dubius* grown in farms near highways [63]. Similar to our results, trace metal(loid)s attained the highest concentrations in the soil nearest the road, and their contents in the sampled plants generally followed this trend [63,64]. In addition to root uptake, foliar uptake of deposited airborne particulate matter is another pathway for trace metal(loid) accumulation in vegetables near roads [65]. To avoid trace metal(loid)contamination in crops resulting from traffic-related emissions, farming soil near busy roads should be monitored

The trace metal(loid) concentrations in the aerial root samples followed the order Zn > Cu > Pb > Sb > Cd (Table 1), and these values were significantly higher than those in the outer bark samples. Compared to the aerial root and outer bark samples, the inner bark samples exhibited the lowest concentrations of trace metal(loid)s (Table 1). The trace metal(loid) concentrations in the aerial roots and bark of the plants at the training site indicated Pb, Zn, Cu, Sb, and Cd concentrations with several times larger magnitudes, namely, 3.1–4.7, 2.8–3.4, 1.9–2.4, 3.0–3.6, and 3.2–4.8, respectively, than those in the control park sample. In comparison with the nearby control park, other than the vehicle training activities, no anthropogenic activities were observed at the venue, and it can be concluded that the excessive amounts of trace metal(loid)s were caused by the additional vehicle emissions.

Considering the sampling locations, the trace metal(loid) concentrations in the tree bark and aerial root samples were heterogeneously distributed at the site and depended on the vehicle activity. Among the five locations (Figure 1 and Table 1), the plant samples at #S1 exhibited the highest concentrations of trace metal(loid)s, including Pb, Cu, Zn, Cd, and Sb. #S1 was located at a ramp near the training site entrance, where the vehicle velocity was rapid and the trainer’s car frequently stopped and started. The rapid and frequent vehicle operation at the hill facilitated the resuspension of large amounts of road dust in the air, which readily deposited on tree bark and leaves [12,51] and resulted in the high accumulation of trace metal(loid)s in plant samples at #S1.

Compared to previous reports, the detected trace metal(loid)s in the outer bark samples exhibited much higher concentrations than those in urban areas in Thailand [66], Toronto, Canada [67], and three European cities [20]. Similar concentrations of Cu, Zn, Cr, and Sb but lower concentrations of Pb, As, and Cd were observed in oak (Quercus) in urban areas in Stassfurt, Germany [19].

Gravitropic aerial roots of banyan trees grow downward from the trunk and can directly sequester airborne particles or absorb nutrients and trace metal(loid) elements from the air. The much higher concentrations of trace metal(loid)s which accumulated in the aerial roots and outer bark can be attributed to their rough layered structure with a large surface area; atmospheric contaminants can be deposited and absorbed in these plant tissues via physiological chemical processes [19]. Tree aerial roots and outer bark can trap atmospheric deposits over many years and subsequently provide more accurate information on the long-term accumulation of atmospheric trace metal(loid)s than tree leaves. The inner bark, which only reflects element uptake from the groundwater and deep soil, can yield the internal background concentration [19]. Compared to our previously reported trace metal(loid) concentrations in banyan tree leaves at the same sampling site in Guangzhou [12], the much higher concentrations of trace metal(loid)s in aerial roots and bark in the current study indicated that these two kinds of tree samples are more sensitive indicators of traffic-related emissions. In addition, the trace metal(loid) uptake capacity through leaf stomata is limited and selective, depending on physiological characteristics, including stomatal density, surface morphology, and structure [68,69], and may not be representative of a large amount of deposited dust particles. To our knowledge, aerial roots were first employed in this field as bioindicators of urban contamination because they exhibit a rough structure with high porosity. Much more accumulation of trace metal(loid)s was found in aerial roots than in bark, suggesting a higher sensitivity of aerial roots for contamination status monitoring. Furthermore, aerial roots are readily available, and sampling them does not affect the tree growth of banyan-like species, in contrast to outer bark and tree leaf sampling. Therefore, aerial roots are more appropriate than bark and tree leaves as bioindicators of urban contamination. 

### 3.3. Sequential Extraction of Trace Metal(loid)s from Dust and Garden Soil

Surface road dust is a direct acceptor of traffic-related emissions; analyzing the chemical availability and fractionation of its trace metal(loid)s can elucidate their probable dissolution or migration from soil to water and organisms. CaCl_2_-extractable elements were investigated using a CaCl_2_ solution and simulated rhizosphere solution [60]. In the CaCl_2_ extract (1/10, g/mL), trace metal(loid)s exhibited a narrow concentration range from not detected (ND) to 388 μg/L, including Zn (61–388 μg/L, <0.47%) and Cu (5–40 μg/L, <0.23%). The simulated organic-based rhizosphere extract (1/10, g/mL) indicated metal concentrations ranging from ND to 1230 μg/L, namely, 5–1230 μg/L (0.4–4.71%) for Zn, 5–88 μg/L (0.21–1.31%) for Cu, and <25 μg/L (<0.03%) for Pb, which were slightly higher than those in the CaCl_2_ extract. The latter values were higher because trace metal(loid) mobility in road dust or soil is generally facilitated by organic exudates released in plant roots [60,70,71]. Hence, vegetables grown near roads could take up water-soluble trace metal(loid)s from road dust, generating adverse effects on food quality and human health.

**Figure 4:**
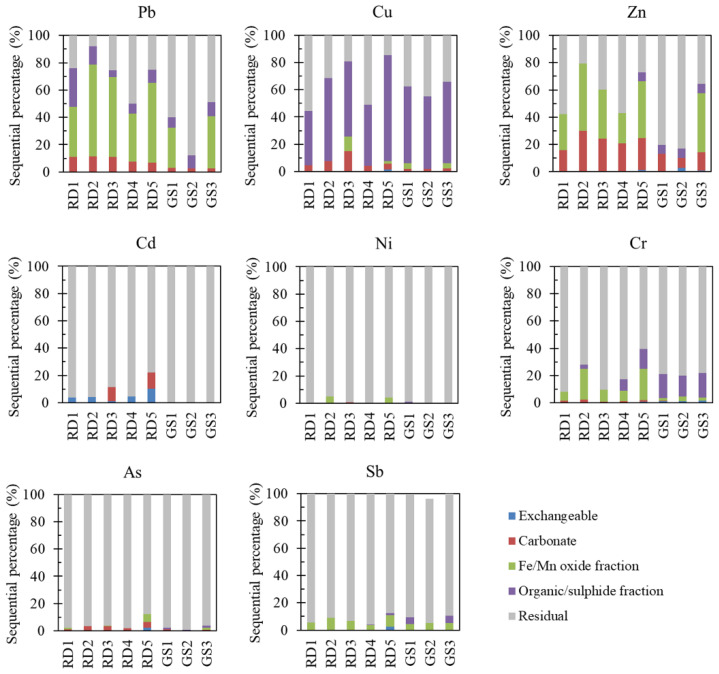
Chemical fractions of trace metal(loid)s in road dust (RD) and garden soil (GS) obtained using the sequential extraction procedure: the exchangeable fraction, carbonate-bound and specifically adsorbed fraction (acid extractable), Fe/Mn oxide fraction (reducible), organic/sulphide fraction (oxidizable), and residual fraction, determined via subtraction from the pseudo-total digestion result.

The other chemical fractions of trace metal(loid)s in the traffic-impacted dust and garden soil samples were further evaluated via sequential extraction to reveal their potential mobility. High carbonate- (F2), Fe/Mn oxide- (F3), and organic/sulphide-associated fractions (F4) were observed for Pb (7–11%, 35–67%, and 5–28%, respectively), Cu (4–15%, 0–10.7%, and 40–78%, respectively), and Zn (16–30%, 22–49%, and 0–6.4%, respectively) (Figure 4). The observed major trace metal(loid)s (Pb, Cu, and Zn) in road dust were generally consistent with previously reported fractions (mean value), such as Pb (12% as F2, 28% as F3, and 16% as F4), Cu (5% as F2, 12% as F3, and 44% as F4), and Zn (21% as F2, 46% as F3, and 11% as F4) in Delhi, India [72]; Pb (70% as F2+F3), Zn (60% as F2, 30% as F3), and Cu (70% as F4) in Hong Kong [1]; and Pb (F45% as F3 and 49% as F5), Zn (36% as F2 and 64% as F5), and Cu (28% as F3, 48% as F4, and 22% as F5) in Nanjing [73]. Anthropogenic traffic-emitted Pb and Zn are commonly precipitated as carbonate minerals or adsorbed by Fe/Mn (oxyhydr)oxides in soil, which can become bioavailable when road dust is exposed to acidic water or dissolved organic substances with an abundance of phenolic hydroxyl and carboxylic groups [38,74]. The appreciable amount of organic/sulphide-associated Cu could be attributed to its higher affinity for organic components [32,72,75] or sulphide minerals [36] in environmental matrices. The first four sequential fractions of trace metal(loid)s in road dust may be released under specific environmental conditions and should be carefully considered.

In contrast to Pb, Cu, and Zn, the majority of Cd (78–96%), Ni (>95%), Cr (61–92%), As (87–98%), and Sb (90–96%) occurred in the residual fractions in the road dust samples (Figure 4). Tire wear-released Sb, Cr, and Ni associated with brake lining are usually found in coarse particles or wear powder [9,14,76], which should be nonlabile. Previous studies have also confirmed the presence of high percentages of the residual fraction of many trace metal(loid)s in street dust, such as Cr (88%), Ni (71%), and Cd (39%) in Delhi, India [72]; and Cd (75%) and Pb (46%) in Asansol, India [29]. The nonlabile residual fractions suggested that these elements in traffic-affected road dust remain stable and are not easily released. Some previous studies reported distinct sequential extraction fractions of Cd in road dust, including ~32% as F2, ~20% as F3, and 15% as F4 in Hong Kong [1]; 41% as F2 in Nanjing [73]; and 27% as F1, 22% as F2, and 39% as F5 in Delhi [72]. Different chemical fractions of trace metal(loid)s have been attributed to their sources and subsequent environmental conditions [73,77]. It is worth noting that Sb and Cd attained high EF values in road dust and should be continuously monitored in terms of their mobile and bioaccessible fractions to prevent potential health risks.

The trace metal(loid)s in garden soils, particularly GS3, showed similar sequential fraction distributions to those in road dust (Figure 4). Considering the high toxicity of Pb, its potential mobility from soil to vegetable plants and its concentration level in vegetables should be noted. GS2 had a much higher residual fraction of Pb (88%), followed by 60% at GS1 and 49% at GS3. The slightly higher residual fraction of trace metal(loid)s in garden soil than in road dust may be attributable to the higher contents of silicate and clay minerals, which would efficiently capture traffic-emitted trace metal(loid)s (particles). Plant growth leads to crucial changes in the pH and organic acid which usually activate the mobilization of trace metal(loid)s and favor uptake by roots [38,78].

How trace metal(loid)s are emitted during traffic activities and how they interact with the road dust matrix are complicated [14,79]. Understanding these processes is critical for evaluating the release behavior of trace metal(loid)s and their resultant risk to the surrounding environment and organisms. Most traffic-emitted contaminants are derived from brake pad wear and tire friction. These emitted trace metal(loid)s usually occur in the form of wear particles, which are most likely difficult to solubilize. Among the samples, RD5 contained the largest exchangeable fractions of trace metal(loid)s, including Zn (10.3 mg/kg, 1.3%), Cu (2.7 mg/kg, 1.6%), Cr (0.8 mg/kg, 0.6%), As (0.3 mg/kg, 2.4%), and Cd (0.1 mg/kg, 10.2%). RD5 was located in the area leading back to the garage where the busiest training activities occurred, including start and stop operations at a slow speed. The results revealed that frequent tire friction and brake wear significantly increased the exchangeable fraction of these toxic metal(loid)s, except for Pb (0.09 mg/kg). The chemical interaction of metal(loid)s (bearing particles) with soil/dust components, including clays, Fe/Mn (oxyhydr)oxides, organic matter, and silicate minerals, as well as their behavior with respect to the soil pH, resulted in their repartitioning [78,80,81]. The low sorption ability/capacity of trace metal(loid)s by dust often results in substantial mobility, and a low content of iron/aluminum oxides and organic matter may also favor trace metal(loid) immobilization [82]. Further identification of traffic-emitted trace metal(loid)s or related particles and their adherence or repartitioning processes within road dust and surface soils still merits further work.

### 3.4. The Implementation of IVG and PBET to Determine the Bioaccessible Trace Metal(loid)s in Road Dust and Garden Soil

**Figure 5:**
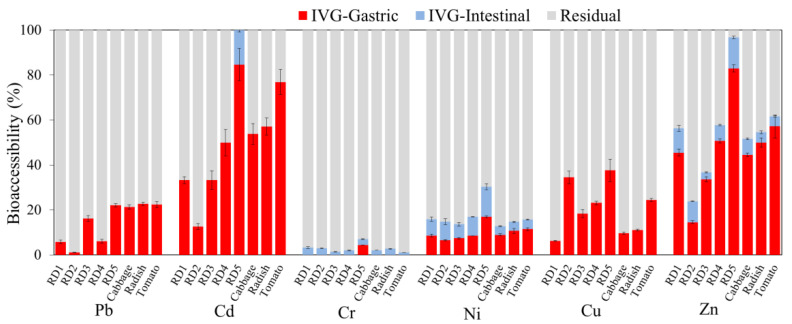
Bioaccessible trace metal(loid)s in the gastric and intestinal phases of the IVG test of road dust and garden soil. Average and standard deviation results of triplicate experiments were plotted. Sb and As in all the samples had bioaccessible fractions lower than 5% and are not plotted.

**Figure 6:**
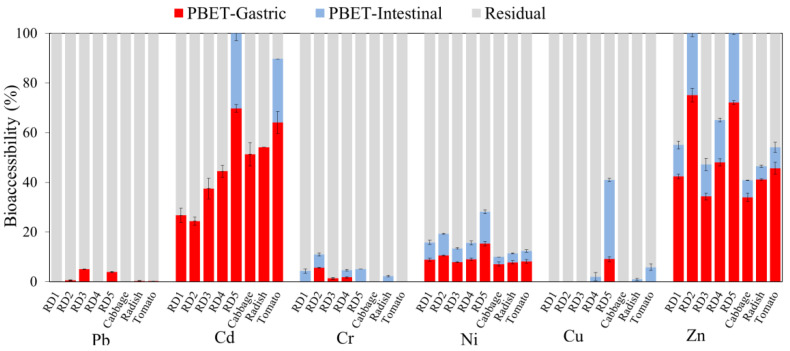
Bioaccessible trace metal(loid)s in the gastric and intestinal phases of the PBET test of road dust and garden soil. Average and standard deviation results of triplicate experiments were plotted. Sb and As in all the samples had bioaccessible fractions lower than 4% and are not plotted.

Trace metal(loid)s in road dust particles often pose health risks to urban residents via incidental ingestion, especially children who typically ingest dust through hand-to-mouth behavior [83]. To reveal this potential risk, it is assumed that only the bioaccessible fractions of trace metal(loid)s can be ingested, rather than the total concentrations, and simulated bioaccessibility tests, including the PBET and IVG, are usually performed [84]. The IVG indicated significantly higher percentages of bioaccessible Pb, Cd, Ni, Cu, and Zn in the gastric phases than in the intestinal phases (*p* < 0.05, Figure 5 and Figure 6), while this comparison is significant in the PBET only for Cd, Ni, and Zn (*p* < 0.05). Higher soluble fractions in gastric phases can usually be explained by the much lower pH values (1.8–2.5), which are conducive to the dissolution of the carbonate- or adsorption-associated fractions of trace metal cations [27,59,85]. The slightly higher Cr in the gastric phase than intestinal phase may be attributed to negatively charged Cr, specifically Cr in the form of CrO_4_^2−^/HCrO_4_^−^, which may be adsorbed at a low pH in the gastric solution and may be released from dust particles to the intestinal solution as the pH rises [86]. 

The dissolved trace metal(loid)s in the gastric and intestinal phases of road dust were summed (Figure 5 and Figure 6), and the results indicated that Pb, Cd, Cu, Ni, and Zn generally included high bioaccessible fractions. A recent study also confirmed that a high proportion of Zn, Cu, Pb, and Cd in urban road dust in northeast Brazil was bioaccessible in simulated gastric and intestinal phases [59]. The results revealed that these trace metal(loid)s may be assimilated in the gastrointestinal tract following incidental soil ingestion, posing a severe health risk. For both the IVG and PBET, the bioaccessible Cd content was significantly higher than the sum of the sequential fractions from F1 to F4, suggesting that some proportion of the typical residual fraction may be released from road dust in the presence of a gastric or intestinal solution. The IVG results indicated that the bioaccessible fractions were the highest for Cd (13–85%) and Zn (14–83%), followed by Cu (6–38%), Pb (1–22%), and Ni (7–17%) (Figure 5). Except for the elevated bioaccessible fractions of Cd and Ni, the bioaccessible contents of trace metal(loid)s determined via the IVG were generally consistent with their potential mobilizable fractions in the sequential extraction results. In contrast to IVG, the PBET results indicated much lower bioaccessible fractions; only Cd (24–69%), Zn (34–72%) and Ni (8–15%) had high fractions, followed by Cu (<9%), Cr (<5%), and Pb (<5%) (Figure 6). The higher soil/solution ratio in the PBET than in the IVG method could result in less mobilization of trace metal(loid)s. The bioaccessible Zn and Ni fractions in the PBET results were consistent with the sequential extraction results (Figure 6); Pb and Cu were substantially underestimated compared to their high potential mobilizable fractions. According to the sequential extraction-determined mobilizable fractions, the IVG gastric simulation showed better performance for the primary contaminants, including Pb, Zn, Cu, and Ni, than the PBET gastric simulation, indicating that the IVG method could be more suitable for evaluating the potential bioaccessibility-related risk of traffic-impacted road dust.

Among the soil and road dust samples, considering the difference in bioaccessible trace metal(loid) fractions in the gastric phase in the IVG test, significantly lower percentages (*p* < 0.01) of Pb, Cd, and Zn were observed in RD2 at #S2, where smooth traffic flow prevails. In contrast, the highest bioaccessible percentages of these three trace metal(loid)s were observed in the road dust from #S5, characterized by frequent start and stop operations. The results indicated the critical role of traffic conditions in the emissions of bioaccessible fractions of Pb, Cd, and Zn.

Notably, consistent with the sequential extraction results, the highly enriched oxyanion trace metalloid Sb was scarcely bioaccessible (<2%). These results suggest that brake wear-released Sb should be highly nonlabile in simulated gastric and intestinal solutions. Nevertheless, a previous study confirmed that Sb extracted from road dust with oxalic acid was dominated by Sb(III) [15], which has a higher toxicity than Sb(V). Sb_2_S_3_ used in brake linings can be oxidized into Sb_2_O_3_ at a high temperature during braking [87] and is easily dissolvable. Although dust components could temporarily sequester vehicle-emitted Sb based on the sequential extraction and bioaccessibility test results, Sb should still be further monitored because brake abrasion dust is the most important source (55.3 ± 7.0%) of Sb pollution in exposed urban areas, particularly areas near intersections [10,88].

### 3.5. Bioaccessibility-Based Health Risk Assessment

**Figure 7:**
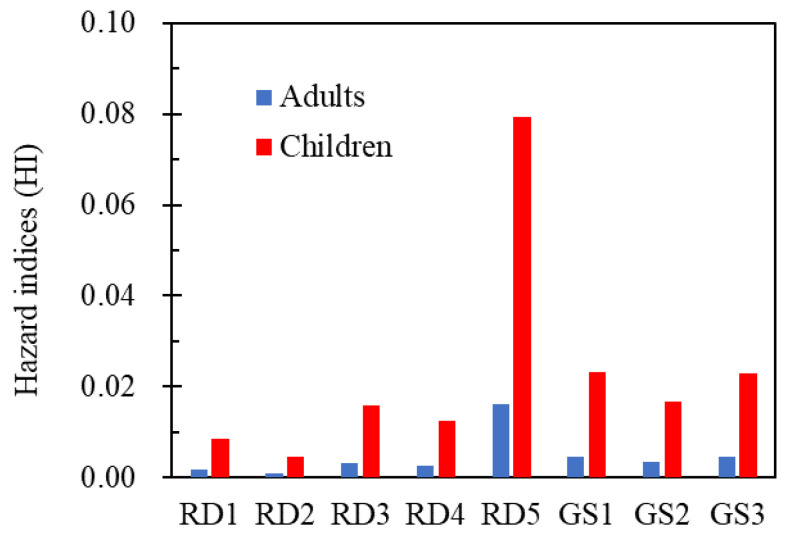
Hazard index (HI) values for the noncarcinogenic effects of ingestion of road dust (RD) and garden soil (GS) in adults and children using bioaccessibility-based health risk assessment.

Because the bioaccessible fractions of trace metal(loid)s determined via the IVG are more reasonable than those determined via the PBET, we employed the IVG bioaccessibility (BA) in the health risk model. The noncarcinogenic HI values of road dust and garden soil ranged from 0.0009 to 0.0161 for adults and 0.0046 to 0.0793 for children (Figure 7), which are lower than one, confirming no necessary concern about noncarcinogenic effects [84]. In the HQ results for noncarcinogenic risk, Pb had major contributions (20–79%, median of 70%), followed by Cr (3–30%, median of 10%) > Zn (5–18%, median of 8%) > Cu (3–23%, median of 5%) ≈ Cd (5–11%, median of 6%), generally consistent with previous findings [26,82]. The results revealed that the potential toxicity of Pb in road dust and roadside soil are still dominant despite the bans on leaded gasoline use, similar to worldwide studies [3,89]. 

The CR results for Pb, Cr, Cd, and Ni ranged from 5 in 10^9^ to 8 in 10^6^ for adults and from 3 in 10^8^ to 1 in 10^5^ for children (Table 2). Although CRs for all the metal(loid)s fall within the range of acceptable threshold values (<10^−4^), a large number of samples showed CR values higher than 10^−6^, including Cd and Ni for adults and Cd, Cr, and Ni for children, suggesting the probable occurrence of carcinogenic health effects from these metal(loid)s in the traffic venue [30,84]. The calculated CRs were more severe than those in previously reported results for urban street dust in Asansol, India [29], and most cities in China [5]. The reason for the higher CRs at the current site is likely attributable to the much higher traffic emissions due to frequent vehicle training activities, including start and stop operations, suggesting the important contribution of vehicle operations to trace metal(loid) contamination of the surrounding environment. 

Fine particles in road dust are readily resuspended due to frequent strong winds and mechanical action [34,35]. Fine dust particles typically contain high contents of toxic elements, such as Pb, Cd, Sb, and Cr [83,90,91], which notably contribute to elevated health risks, particularly for children. Therefore, the evaluation of human health risks based on bulk road dust could be underestimated.

In summary, road dust at the study traffic-training venue poses a probable health risk to the instructors and trainees. Currently, a primary school is under construction at the site, and the workers should be aware of incidental ingestion. Furthermore, to reduce the possible health risk to children playing nearby, the road dust and surface soil at the venue should be carefully managed via appropriate remediation or safe disposal. Based on our work, road dust in urban parks near busy roads may expose humans to health risks.

## 4. Perspectives

Our study confirmed that road dust in areas with frequent stops and starts, such as parking garages and city intersections, where not only total concentrations of trace metal(loid)s but also bioaccessible fractions are high, should be carefully assessed in future work. The trace metal(loid)s in road dust usually originate from materials associated with deceleration operations, such as tire wear, brake lining, and even petrol combustion residues. A recent study further confirmed that very high concentrations of Pb and Cr in roads may originate from lead chromate used as a pigment in colored road makings in European countries [92]. Nevertheless, the contributions of these potential sources to trace metal(loid) accumulation in megacities still remain unclear, particularly for some previously overlooked metal(loid)s, including Sb and Cd. The chemical speciation and partitioning of trace metal(loid)s from original traffic-associated sources to the environment largely depend on the source characteristics and soil or dust components. Future elucidation of the trace metal(loid) redistribution process combining the newly-developed stable isotopes technique [14,89] and synchrotron X-ray absorption spectroscopy [8,36,93] should provide critical valuable information on their source identification, mobility, and bioaccessibility in road dust and soil.

## 5. Conclusions

This study presented a comprehensive characterization of the distribution, sequential fraction, and bioaccessibility of trace metal(loid)s in road dust and garden soil and their transferability from soil to plants at a traffic-training venue. The results indicated high EF values for Cd, Sb, Zn, Cu, and Pb in road dust; the highest concentrations were observed in the area leading to the garage due to increased tire and brake pad lining wear resulting from the higher vehicle volume and more frequent vehicle starts and stops. The aerial roots of local banyan trees exhibited the highest Pb, Zn, and Sb levels under hill start conditions, possibly caused by high road dust resuspension due to higher velocities and more frequent braking and stops and starts; hence, aerial roots are an appropriate bioindicator. Consistently, the concentrations of trace metal(loid)s in garden soil at the venue and of Pb and Cd in leafy vegetables exceeded the permissible recommended values. Integrated chemical sequential extraction and bioaccessibility tests demonstrated that the potential mobilizable Pb, Cd, and Ni may pose human health risks. Therefore, this vehicle training venue is an appropriate site to evaluate the geochemistry and health risk of trace metal(loid)s in road dust originating from specific vehicle operations, and the obtained information is crucial for the reutilization of similar contaminated sites. 

## Figures and Tables

**Table 1:** Concentrations of Fe and Al (% in road dust and garden soil: mg/kg in plant samples), Mn (mg/kg), and trace metal(loid)s (mg/kg) in road dust, garden soil, vegetables, aerial roots, outer bark, and inner bark of banyan trees in five sampling locations (#S1–5, with details shown in Figure 1) at the traffic-training venue. Radish leaf, cabbage leaf, and tomato fruit are the edible part, while tomato leaf is the non-edible residue in the field.

Matrix	Sample	Fe	Al	Mn	Pb	Cd	Cr	Ni	Cu	Zn	Sb	Ba	As
Road dust (n = 5)	RD1	2.67	5.05	655	77.4	0.42	59.0	17.0	70.0	269	4.28	350	15.7
	RD2	1.83	4.24	453	91.0	0.71	47.1	13.4	41.7	207	2.51	290	8.4
	RD3	2.68	4.67	901	88.3	0.48	71.2	17.3	61.2	230	3.59	450	20.2
	RD4	2.32	4.41	516	114	0.48	68.1	17.3	78.9	395	6.30	380	14.2
	RD5	2.00	3.38	386	282	0.96	128	25.3	172	818	8.64	490	14.5
	Control park	1.87	2.67	361	35.9	0.32	60.0	12.5	26.8	139	2.10	240	8.9
Garden soil (n = 3)	GS1: Radish	2.95	7.30	268	94.4	0.35	67.2	17.4	78.0	261	3.18	290	31.4
	GS2: Cabbage	3.71	7.65	198	70.8	0.26	74.1	19.1	67.2	205	2.75	250	40.3
	GS3: Tomato	3.00	6.90	348	96.3	0.26	64.5	15.9	74.0	230	2.90	270	32.5
Soil	Deep soil (20–25 cm) in the venue	4.25	8.83	219	77.7	0.05	81.1	15.9	31.7	38	0.67	160	20.6
Soil	Guangzhou red soil [50]			132	34.4	0.03	43.2	13.0	14.4	49			10.5
Vegetables (n = 5)	GS1: Radish leaf	874	520	195	4.32	0.64	5.1	2.8	18.1	322	1.18	55.5	0.84
	GS2: Cabbage leaf	820	500	104	4.42	0.73	6.5	13.0	13.2	232	1.23	59.6	0.87
	GS3: Tomato leaf	1240	460	140	11.0	1.33	7.7	3.4	43.6	162	3.61	77.0	1.29
	GS3: Tomato leaf-2	2080	860	192	13.0	1.61	6.2	2.8	52.8	111	1.52	106	2.41
	GS3: Tomato fruit	53	10	12.4	0.13	0.10	0.6	0.4	11.6	28	0.01	1.2	0.02
Aerial root (n = 6)	#S1	2880	830	71.9	52.4	1.16	36.6	32.2	113	577	2.68	23.3	0.78
	#S2	918	340	32.1	17.2	0.42	16.4	8.8	55.1	217	1.51	74.5	0.52
	#S3	851	290	30.4	26.1	0.64	51.8	27.9	62.3	217	1.52	58.0	0.48
	#S4	680	210	30.2	17.2	0.57	7.2	5.6	49.7	223	1.43	57.1	0.43
	#S5	736	260	27.4	19.1	0.45	10.6	6.0	52.5	182	1.26	50.5	0.51
	Control park	331	190	10.7	5.6	0.13	6.9	2.6	25.7	65	0.42	23.3	0.31
Outer bark (n = 5)	#S1	1960	650	128	24.1	0.70	14.4	12.1	82.5	344	1.49	102	0.78
	#S2	93	40	50.6	2.1	0.12	1.3	3.7	9.9	53	0.07	65.3	0.06
	#S3	371	130	71.6	9.2	0.25	3.9	3.8	25.1	102	0.77	53.7	0.20
	#S4	309	110	46.2	5.2	0.16	4.3	5.7	14.6	69	0.24	72.4	0.18
	#S5	143	50	24.9	3.3	0.14	1.5	5.4	9.8	46	0.11	42.0	0.18
Inner bark (n = 5)	#S1	96	320	16.4	0.7	0.03	2.4	3.9	4.6	14	0.07	21.4	0.07
	#S2	420	30	11.7	0.2	0.01	86.9	5.8	2.4	2.7	0.01	55.5	0.04
	#S3	33	10	7.2	0.2	0.02	1.5	0.7	2.0	3.7	0.02	29.3	0.06
	#S4	32	10	5.6	0.2	0.01	2.7	3.2	1.6	4.3	0.02	54.1	0.06
	#S5	35	130	4.2	0.2	0.02	3.4	10.7	2.0	4.8	0.02	37.2	0.10

**Table 2:** Cancer risk (CR) values for Pb, Cd, Cr, and Ni in road dust (RD1–5) (n = 5) and garden soil (GS1–3) (n = 3) using bioaccessibility-based health risk assessment at the traffic-training venue. The number of samples for non-negligible CR values of 10^−6^ and 10^−4^ for each metal are listed.

Samples	Adults				Children			
	Pb	Cd	Cr	Ni	Pb	Cd	Cr	Ni
RD1	2 × 10^−8^	1 × 10^−6^	6 × 10^−7^	1 × 10^−6^	1 × 10^−7^	6 × 10^−6^	3 × 10^−6^	7 × 10^−6^
RD2	5 × 10^−9^	8 × 10^−7^	4 × 10^−7^	1 × 10^−6^	3 × 10^−8^	4 × 10^−6^	2 × 10^−6^	5 × 10^−6^
RD3	7 × 10^−8^	1 × 10^−6^	3 × 10^−7^	1 × 10^−6^	4 × 10^−7^	7 × 10^−6^	1 × 10^−6^	6 × 10^−6^
RD4	4 × 10^−8^	2 × 10^−6^	4 × 10^−7^	2 × 10^−6^	2 × 10^−7^	1 × 10^−5^	2 × 10^−6^	8 × 10^−6^
RD5	3 × 10^−7^	8 × 10^−6^	3 × 10^−6^	4 × 10^−6^	2 × 10^−6^	4 × 10^−5^	1 × 10^−5^	2 × 10^−5^
GS1	1 × 10^−7^	2 × 10^−6^	6 × 10^−7^	1 × 10^−6^	5 × 10^−7^	9 × 10^−6^	3 × 10^−6^	7 × 10^−6^
GS2	8 × 10^−8^	1 × 10^−6^	5 × 10^−7^	1 × 10^−6^	4 × 10^−7^	6 × 10^−6^	2 × 10^−6^	6 × 10^−6^
GS3	1 × 10^−7^	2 × 10^−6^	2 × 10^−7^	1 × 10^−6^	5 × 10^−7^	9 × 10^−6^	1 × 10^−6^	7 × 10^−6^
n (10^−6^~10^−4^)	0	5	1	2	1	8	6	8

## Data Availability

The associated dataset of the study is available upon request to the corresponding author.

## Supplementary Materials

The following supporting information can be downloaded at: https://www.mdpi.com/article/10.3390/ijerph20032520/s1, ICP-MS and MP-AES analysis details; Table S1: Summary of the IVG and PBET bioaccessibility experimental details; Table S2: Parameters of the chronic daily intake equation in the human health risk model. References [94,95] are cited in the supplementary materials.
