# Supplementary material for: Trace Metal(loid) Migration from Road Dust to Local Vegetables and Tree Tissues and the Bioaccessibility-Based Health Risk: Impacts of Vehicle Operation-Associated Emissions
Source: Int J Environ Res Public Health. 2023 Jan 31;20(3):2520. doi: 10.3390/ijerph20032520 (PMC9914983; doi:10.3390/ijerph20032520)
Supplement: Supplementary file 1 [file ijerph-20-02520-s001.zip › ijerph-2140891-supplementary.pdf]

Supplementary Materials

Trace metal(loid) migration from road dust to local vegetables and tree tissues and bioaccessibility-based health risk: Impacts of vehicle operation-associated emissions

Guangbo Liu [1, 2], Tian Chen [3], Jinli Cui [1, 2*], , Yanping Zhao [4], Zhi Li [1], Weixin Liang [4], Jianteng Sun [2], Zhenghui Liu [2], Tangfu Xiao [1]

1 Key Laboratory for Water Quality and Conservation of the Pearl River Delta, Ministry of Education, School of Environmental Science and Engineering, Guangzhou University, Guangzhou 510006, China

2 Guangdong Provincial Key Laboratory of Petrochemical Pollution Processes and Control, School of Environ-mental Science and Engineering, Guangdong University of Petrochemical Technology, Maoming 525000, China

3 Department of Civil and Environmental Engineering, The Hong Kong Polytechnic University, Hung Hom, Kowloon, Hong Kong

4 Guangdong Provincial Key Laboratory of Chemical Measurement and Emergency Test Technology, Institute of Analysis, Guangdong Academy of Sciences (China National Analytical Center, Guangzhou), Guangzhou 510070, China

* *Correspondence: jlcui@gzhu.edu.cn*

ICP-MS and MP-AES analysis details:

ICP-MS analysis details are as follows. The radiofrequency power was 1.55 kW, and the gas flow was 15 L/min for plasma, 0.99 L/min for nebulization, and 0.9 L/min for auxiliary gas. For kinetic energy discrimination (KED), He was used at 4 mL/min. The isotopes ^{56}Fe, ^{27}Al, ^{208}Pb, ^{111}Cd, ^{52}Cr, ^{60}Ni, ^{63}Cu, ^{66}Zn, ^{55}Mn, ^{121}Sb, ^{137}Ba, and ^{75}As were used for identification and quantification, while In solution was used as the internal standard. The MP-AES operating details for Fe and Al analysis were as follows. The standard sample introduction system comprised of a concentric nebulizer of 240 kPa, rinse time of 10 s, stabilization time of 10 s, and background correction using auto. The atomic lines were 259.026 nm for Fe and 369.152 nm for Al.

Table S1. Summary of the IVG and PBET bioaccessibility analysis results.

Method	Extraction phase	Composition (g L^{-1})	Solid/solution ratio	pH	Extraction time (h)
IVG	Gastric	10 g pepsin, 8.77 g NaCl	1:150	1.8	1
	Intestinal	3.5 g bile, 0.35 g pancreatin	1:150	5.5	1
PBET	Gastric	1.25 g pepsin, 0.5 g sodium malate, 0.5 g sodium citrate, 420 μL lactic acid, 500 μL acetic acid	1:100	2.5	1
	Intestinal	1.75 g bile, 0.5 g pancreatin	1:100	7.0	4

Table S2. Parameters of the chronic daily intake equation in the human health risk model.

Parameter	Definition	Unit	Values		Reference
			Adults	Children	
C	Trace metal concentration	mg/kg	-	-	-
BA	Bioaccessibility	%	-	-	-
IR	Ingestion Rate	mg/d	100	200	(MEP, 2014)
ExFre	Exposure Frequency	day/year	350	350	(MEP, 2014)
ED	Exposure Duration	year	25	6	(MEP, 2014)
AT	Average time	days	Non-carcinogenic 26280<br>Cancer 9125		(MEP, 2014)
BW	Average body weight	kg	56.8	15.9	(MEP, 2014)
RfD	Reference dose	mg/kg/d	Note #1		(USEPA, 2010; 2013).
SF	Slope Factor	1/(mg/kg/d)	Note #2		(USEPA, 2010).

Note #1: RfD (mg/kg/d) is 0.0035 for Pb and 0.3 for Zn. Note #2: SF (1/(mg/kg/d)) is 0.0085 for Pb.

References

MEP, 2014. Technical Guidelines for Risk Assessment of Contaminated Sites. HJ 25.3-2014. Ministry of Environmental Protection of China

USEPA, 2010. Regional Screening Levels. Available online at http://www.epa.gov/region9/superfund/prg/index.html.

USEPA, 2013. Region IX, regional screening levels (formerly PRGs). Vol. CA 94105.
